# Long-term monitoring reveals invariant clutch size and unequal reproductive costs between sexes in a subtropical lacertid lizard

**DOI:** 10.1186/s40851-019-0152-0

**Published:** 2020-01-06

**Authors:** Jhan-Wei Lin, Ying-Rong Chen, Tsui-Wen Li, Pei-Jen L. Shaner, Si-Min Lin

**Affiliations:** 1 School of Life Science, National Taiwan Normal University, No. 88, Sec. 4, Tingzhou Rd., Taipei, Taiwan; 2 Department of Biology, National Museum of Natural Science, No. 1, Guanqian Rd., Taichung, Taiwan

**Keywords:** *Anolis*, Capture-mark-recapture, Female-bias mortality, Gekkonidae, Sexual dimorphism, Survival

## Abstract

Based on 20,000 records representing *c*. 11,000 individuals from an 8-year capture-mark-recapture (CMR) study, we tested and confirmed a new case of invariant clutch size (ICS) in a sexually dichromatic lacertid lizard, *Takydromus viridipunctatus*. In the grassland habitat of the early succession stage, females showed strictly low and invariant clutch size, multiple clutches in a breeding season, high reproductive potential, and annual breeding cycles that correspond to the emergence of male courtship coloration. The hatchlings mature quickly, and join the adult cohort for breeding within a few months, whereas adults show low survival rates and a short lifespan, such that most die within one year. Mortality increased in both sexes during the breeding season, especially in females, indicating an unequal cost of reproduction in survival. These life history characters may be explained by two non-exclusive hypotheses of ICS—arboreal hypothesis and predation hypothesis—within the ecological context of their habitat. Our study highlights a confirmed case of ICS, which adapts well to this *r*-selected grassland habitat that experiences seasonal fluctuation and frequent disturbance.

## Background

Life history traits and reproductive strategies are keys to our understanding of demographic processes and population dynamics in ecological studies. Natural selection predicts that organisms should optimize the timing of reproduction (e.g. age or season) and the number of offspring in a single reproductive event (e.g. clutch size) in order to maximize their fitness [1–3]. However, specific ecological conditions can add constraints to such optimization. For instance, an environment with high predation risks can favor early sexual maturation and a large clutch size in one or a few breeding events [4], while simultaneously adding constraints on the inter-birth intervals and how large the clutch size can be, given the poor physiological condition of food-limited breeders. The tradeoff between survival and reproduction under resource limitation, also known as “cost of reproduction” [5–7], is dynamic and can differ significantly across species and ecosystems, leading to variation in the optimized clutch size.

Vertebrate clutch size is either variable (VCS; variable clutch size) or invariant (ICS; invariant clutch size). Variation in clutch size for VCS species is usually condition-dependent, influenced by intrinsic factors such as age, body size, or nutrition condition, and/or external factors such as resource abundance [6, 8–10]. In contrast, ICS species consistently produce one or two offspring per clutch. VCS is thought to be the ancestral state of reptiles, occurring in crocodiles, tuataras, most turtles, and most squamate taxa [11, 12]. ICS independently evolved several times in lizards, and is widespread among anoles [13] and gekkotan [14] lizards.

Several nonexclusive hypotheses have been proposed to explain the advantages of the ICS strategy in the following conditions [15, 16]. The “arboreal hypothesis” [15] indicates that lizards evolved to reduce their clutch size due to the necessity of perching on plants (e.g., *Anolis* spp.) or the smooth surfaces (gekkonid lizards). The “predation hypothesis” [9, 15] in contrast proposes that females separate their clutch in the breeding season to reduce predation risks. A third hypothesis proposes that ICS may have evolved to increase the size of the eggs and thus create high-quality offspring [17, 18]. Introduced by MacArthur and Wilson [19], the *r*-selected and K-selected strategies represent the traits of large clutch size with low offspring quality and small clutch size with high quality, respectively [2, 20]. Based on the third hypothesis, evolution of ICS may also be closely associated with the K-selected reproductive strategy.

Comprehensive studies of life history strategies, population dynamics, and how they relate to selection pressures require precise estimates of several parameters. These include life history traits, such as age at sexual maturity, fecundity, and clutch size; and demographic traits such as growth rate, survival probability, and population size [2, 3]. Acquiring such information is challenging in wild populations, leading to the lack of empirical studies on links between life history and demography [21]. One way to obtain these estimates is to track a sufficient number of individuals within a population for a period of time, such as the capture-mark-recapture (CMR) approach [22]. Because an ideal CMR system requires a high recapture rate, species with limited mobility in a relatively isolated habitat should be better suited for CMR studies (compared to species with high mobility or in open populations). However, despite the rapid developments in statistical theories and computation tools in the field of CMR studies, it remains extremely labor-intensive to conduct long-term CMR studies on wild vertebrate populations. Until now, CMR studies with sufficient sample sizes are comparatively rare.

**Fig. 1:**
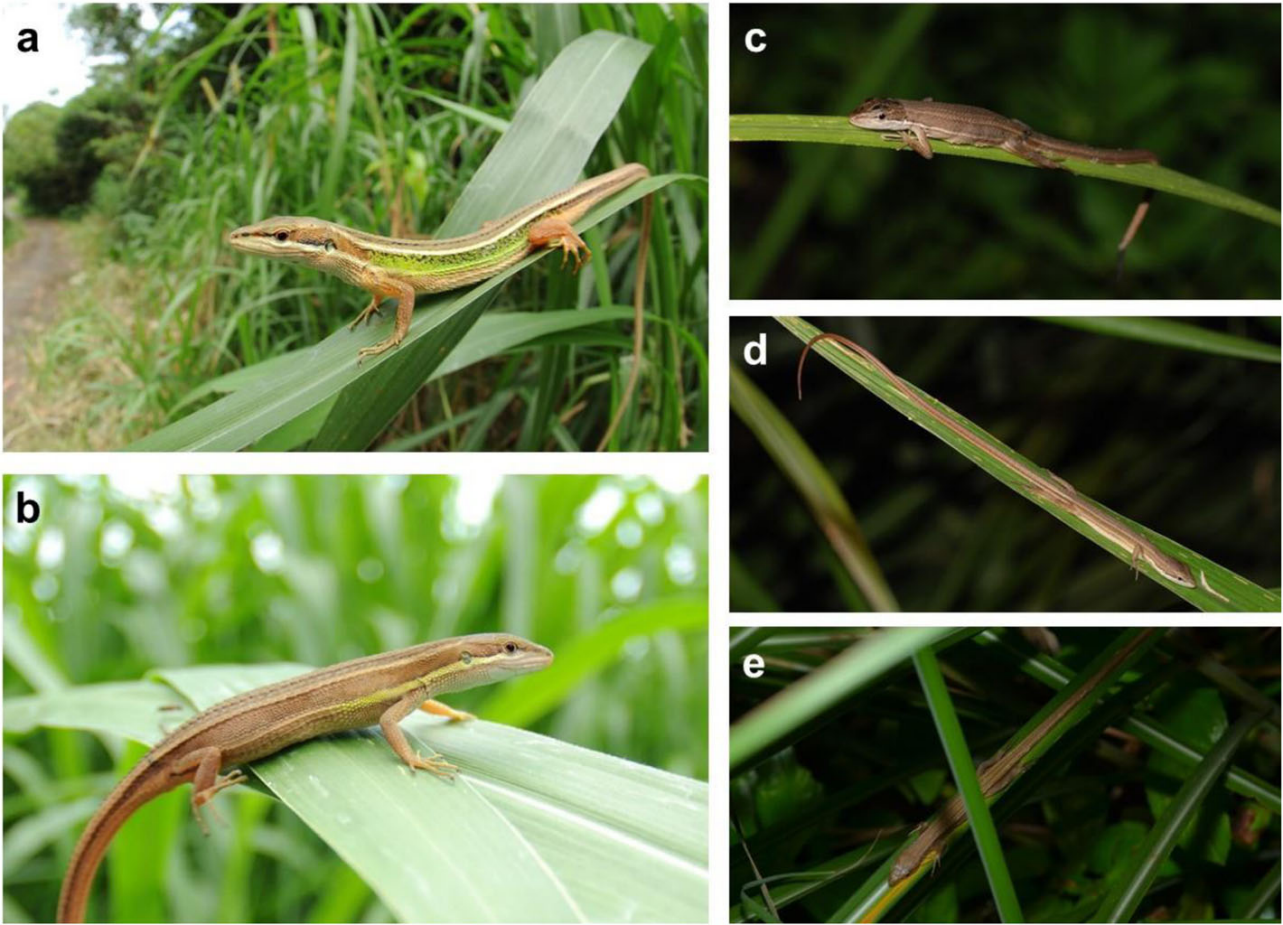
*Takydromus viridipunctatus* is characterized by its greenish nuptial color on males (**a**) in the breeding season. In contrast, females (**b**) are brownish all year round. The specialized perching behavior on the grasses (**c**, **d**, **e**) makes them an excellent target for testing the “arboreal hypothesis” of invariant clutch size (ICS)

In the present study, we aimed to confirm a new case of ICS in a grass-perching lizard inhabiting a subtropical grassland habitat under frequent disturbances. *Takydromus*, also known as grass lizards, is a group of small-sized lacertid lizards comprising about 20 species distributed in Oriental and eastern Palaearctic regions [23, 24]. Most *Takydromus* species adapt open grasslands in the early succession stage; some achieve extremely high local population densities, making them an idea model species for demographic studies. Based on past research, *Takydromus* lizards have ZZ/ZW sex chromes [25], and the same sex determination is widely shared across lacertids [26]. *Takydromus viridipunctatus* Lue and Lin, 2008, the green spotted grass lizard (Fig. 1), is a sexually dichromatic species which may be the most intensively studied among congeners with respect to ecology and evolutionary biology [27–31]. During the breeding season, adult males exhibit intense green spots on their lateral body as nuptial coloration, while females remain dull brown year-round.

By selecting *T. viridipunctatus* as the target species, this study can fill several knowledge gaps in life history studies of squamate reptiles. First, most studies relevant to ICS concentrated on the two major taxa, i.e., *Anolis* and gekkotan lizards [15, 32, 33]; knowledge of other taxa is much more limited. Secondly, long-term demographic studies on reptile species are scarce and biased towards temperate regions in Europe and North America [34]; a demographic study from this species could help establish a model for the seasonal fluctuation of reptiles in this subtropical region. The third goal is to show that the reproductive trait of ICS adapts well to the life history of high breeding potential in the habitat of early succession stage, which is usually regarded as an *r*-selected environment. The fourth and final goal is to show the significant decrease and distinct difference between males and females in their monthly survival rates, which may demonstrate the consequence of “cost of reproduction” in sexually dichromatic species.

From 2006 to 2013, we conducted a monthly CMR study of a relatively closed *T. viridipunctatus* population in a cape on the northern coastline of Taiwan. Given that the cape environment is frequently and intensely disturbed, and that *T. viridipunctatus* is small-sized, grass-perching species (Fig. 1), we hypothesized that this lizard should show life history traits such as fast maturity, low survival rate, and short life span, but high breeding potential. For survival cost of reproduction, we further asked whether there is a difference between males and females in this sexually dichromatic species. By accumulating a large number of CMR records, our results provide evidence for ICS in this grass lizard, along with multiple life history traits which are adaptive to a grassland habitat under high disturbance.

## Methods

### Study system

From 2006 to 2013, we monitored a population of *T. viridipunctatus* in Shitoushan Park, Jinshan Cape, northern coast of Taiwan (25°13′34″N, 121°38′55″E). The area has a subtropical monsoon climate (annual precipitation: 3772 mm) with four distinct seasons: spring from March to May (mean temperature = 21.2 °C), summer from June to August (mean = 28.5 °C), autumn from September to November (mean = 24.1 °C), and winter from December to February (mean = 16.4 °C). The cape is surrounded by ocean and urban areas, hence the emigration and immigration rates could be neglected and the population could be assumed as a closed population. The primary causes of mortality for this lizard population are avian predation [30] and parasitism by trombiculid mites (*Leptotrombidium* sp.) [27]. Other potential predators, such as carnivores and snakes, are uncommon in this area.

### Capture-mark-recapture (CMR) survey

The CMR survey of *T. viridipunctatus* was conducted one night per month from May 2006 to August 2013. On the night after a sunny day in the beginning of every month, seven to twelve experienced fieldworkers searched several transects totaling 800 m in length for 2–3 h. All individuals encountered were hand-captured from their sleeping perch on the grasses or herbs. The captured lizards were weighed to the nearest 0.01 g using an electronic scale, and their snout-vent lengths (SVL) measured to the nearest 0.01 mm using a digital caliper. Sex was recorded according to the presence/absence of lateral green spots and the lump of hemipenes in the base of the tail. Small individuals without obvious sign to determine sex were designated juveniles. The number of trombiculid mites on each lizard was counted, and the condition of reproduction and autotomy were recorded (see below). All captured individuals were uniquely tagged by toe clipping. The individuals were released back to the study site after processing. A previous study suggested that there was no systematic bias in capture probability between sexes when using the capture procedure in this system [27].

### Evaluation of reproductive condition

The nuptial green spots, which appear only on males and are most intense during breeding season, may play an important role in their courtship behavior [24, 31]. Herein we used a “nuptial index”, the coverage ratio of the lateral green spots, to indicate the amount of reproductive effort for males. This parameter included three categories: dull males with no green spots on lateral side as level one, medium males with < 40% lateral green coverage as level two, and bright males with > 40% lateral green coverage as level three. For females, we used bite scars and pregnancy to indicate their reproduction effort. The bite scars are from the biting of the males during copulation, and the number of bite scars reflect the number of copulations a female recently experienced. We scored no bite scar as the lowest level of copulations; one bite scar as the medium level; and more than two bite scars as the highest level. Pregnancy and number of eggs were evaluated by abdominal palpation technique [35], which is the most common method for inspecting the reproduction situation in lizards without invasive treatments. Females without eggs were assigned as non-pregnant; those with eggs were assigned as pregnant and the number of eggs was recorded.

### Survival estimation

We applied the Cormack-Jolly-Seber (CJS) model as implemented in program MARK to estimate adult survival rate. The CJS model allows us to simultaneously estimate the probability of any given individual remaining in the population between capture occasions (apparent survival, *φ*) and capture probability (*p*) at each capture occasion [36]. For an assumed closed population, the former is the survival rate that we want to estimate. The latter is the probability for an individual to be detected, which is an essential derivative in this model to prevent bias in survival estimation caused by detectability. This model allows the incorporation of predicted variables for both apparent survival and capture probability. The models of different predictors for these two probabilities are comparable by using model selection based on corrected Akaike’s Information Criterion (AICc). The significance of these predictors was examined by likelihood ratio tests (LRT). In CJS model, although apparent survival is influenced by survival and emigration, our study site was surrounded by urban areas and the ocean, making emigration less probable. Therefore, the influence of emigration in our survival estimation is likely negligible. Since juveniles may have different life-history parameters and detectability from those of adults, non-adult records were not used in survival estimation.

We evaluated the capture probability first to find its best supported form because this step could largely simplify the complexity of the modelling by squarely reduce the number of models in model selection. The sex- and time-varied capture probability (*p*_sex*time_, time: monthly varied) was best supported in this step. We then used *p*_sex*time_ to construct seven models of apparent survival (Table 1): 1) constant survival model (*φ.*): survival is a fixed value that did not change with sex, season, or time; 2) sex-varied survival model (*φ*_sex_): survival is sex-specific; 3) season-varied survival model (*φ*_season_): survival varied with biological seasons (breeding season from May to October; non-breeding season from November to April); 4) time-varied survival model (*φ*_time_): survival varied with every capture occasion; 5) sex- and season-varied with interaction model (*φ*_sex*season_): sex-specific survival varied with biological seasons allowing males and females to have different patterns; 6) sex- and season-varied without interaction (*φ*_sex + season_): sex-specific survival varied with biological seasons but both sexes had the same pattern; and 7) sex- and time-varied with interaction (*φ*_sex*time_): sex-specific survival varied with every capture occasion, which allows males and females to have different patterns. These seven models were compared in the model selection according to AICc. The significance of the predictors, time, sex, season, and the interactions, were examined by LRT. The full model (*φ*_sex*time_, *p*_sex*time_) had an acceptable model fit (RELEASE GOF: χ^2^ = 279.07, df = 307, *P* = 0.8721; bootstrap GOF: c-hat = 0.7028), suggesting that the reduced models would not be over-parameterized.

**Table 1:** The model selection of seven candidate models for adult apparent survival

Model	AICc	∆ AICc	Weights	Model Likelihood	No. Par	Deviance
*φ*_sex*season_ *p*_sex*t_	11,560.55	0.00	0.46	1.00	82	2160.46
*φ*_season_ *p*_sex*t_	11,561.64	1.09	0.27	0.58	80	2165.67
*φ*_sex + season_ *p*_sex*t_	11,561.85	1.30	0.24	0.52	81	2163.83
*φ*_._ *p*_sex*t_	11,567.40	6.86	0.02	0.03	79	2173.50
*φ*_sex_ *p*_sex*t_	11,567.73	7.18	0.01	0.03	80	2171.76
*φ*_t_ *p*_sex*t_	11,582.15	21.61	0.00	0.00	111	2122.00
*φ*_sex*t_ *p*_sex*t_	11,595.96	81.80	0.00	0.00	156	2087.70

sex: males and females; season: breeding season (May–October) and non-breeding season (November–April); t = month;. =constant

## Results

### The 8-year CMR data set

The 8-year CMR data set contained 20,552 records from 11,415 unique individuals over 88 monthly capture occasions. Monthly mean of total captures over this period was 233 individuals, but exceeded 500 in some occasions. The captured individuals fluctuated with a distinct annual cycle; lowest in February and highest in October and November (Additional file 1: Figures S1a and S1b). Except for the first several months, the recapture rate (the ratio of recapture individuals in each capture event; ranging between 0.20 and 0.93) was highest in June and July (0.6–0.9) and lowest in October (0.2–0.3; Additional file 1: Figure S1c).

### Population structure and sexual dimorphism

During the study, there were 10,868 captures of 4504 adult individuals, including 5692 captures of 2375 males and 5176 captures of 2129 females. The dynamics of captured individuals were showed in Additional file 1: Figures S1a and S1b. The sex ratio fluctuates with a regular annual pattern; male-biased in winter and spring, and female-biased in summer (Additional file 1: Figure S1d). The combined sex ratio is 1.08:1 (male:female), which is significantly male-biased (Chi square test: χ^2^ = 24.70, df = 1, *P* < 0.0001).

The smallest male showing lateral green spots was 36.19 mm in SVL, and the smallest pregnant female was 37.89 mm. Males (*N* = 2375) and females (*N* = 2129) were similar in SVL (Wilcoxon rank-sum test: χ^2^ = 2.71, df = 1, *P* = 0.0996; Additional file 1: Figure S2a), with median SVL for the males and females being 46.58 mm and 47.38 mm respectively. However, males had a higher body weight than females (Wilcoxon rank-sum test: χ^2^ = 61.49, *P* < 0.0001; Additional file 1: Figure S2b), with the median body weight for the males and females being 1.92 g and 1.83 g, respectively. The increase of mass difference between sexes following the increase of SVL was significant (multiple regression analysis with log-transformation in both weight and SVL: *R*^2^ = 0.7969, F_3,4500_ = 5886.72, *P* < 0.0001; sex: *F*_1,4500_ = 6.56, *P* = 0.0105; SVL: *F*_1,4500_ = 6538.44, *P* < 0.0001; sex×SVL: *F*_1,4500_ = 9.69, *P* = 0.0019; Additional file 1: Figure S2c).

### Annual breeding season and invariant clutch size

**Fig. 2:**
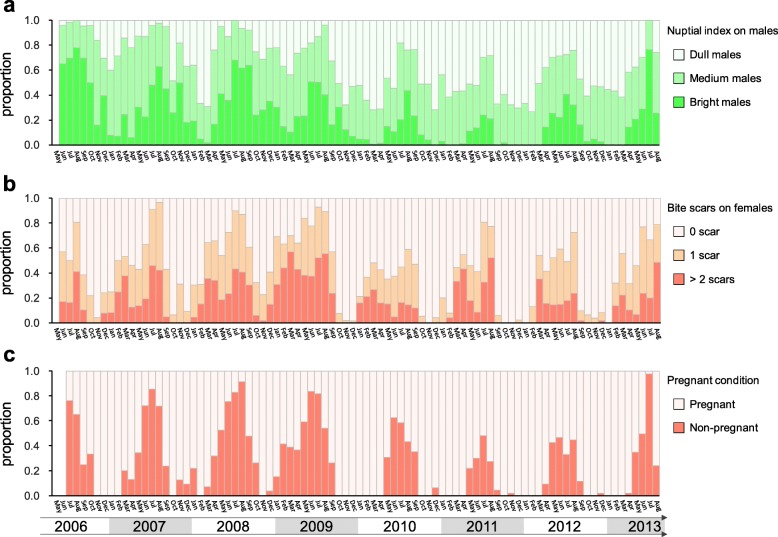
The annual reproduction cycles of *Takydromus viridipunctatus* based on the eight-year capture data during 2006–2013. The reproduction efforts were represented by three indexes: (**a**) the proportion males with bright, medium, or dull nuptial color, categorized by the coverage of green areas on the lateral sides; (**b**) the proportion of females with different levels of copulation intensity, categorized by the number of bite scars; (**c**) the proportion of pregnant and non-pregnant females

The reproduction of the lizard follows an annual cycle; the breeding season lasts from May to October every year. This observation is based on the seasonal pattern of nuptial index in males (Fig. 2a), and the number of bite scars and pregnancy index in females (Fig. 2b and c). The clutch size ranges from one to three, with a mean of 1.55. Among the 1709 pregnant females, 810 (47.4%) have one egg, 870 (50.9%) have two, and 29 (1.7%) have three. With more than 98% of the females producing less than two eggs, the clutch size of this lizard closely fits the definition of ICS [11, 12]. Females laid multiple eggs in the breeding season; a total of 214 recaptured females were recorded as pregnant at least twice in the same breeding season. The highest number of pregnancies we recorded from a single female was five.

### Annual demographic structure and age to sexual maturity

**Fig. 3:**
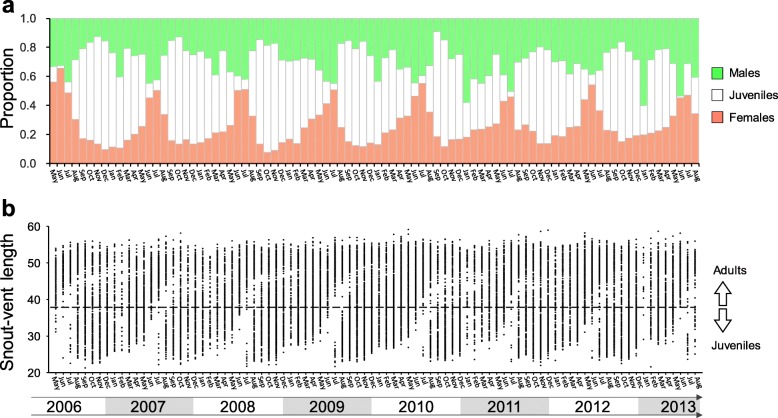
The annual penetration cycles from juveniles to adults based on the eight-year capture data during 2006–2013. **a** Usually in August of every year, a notable amount of neonates begins to hatch, and reaches the highest proportion of the population from September to December. In the next spring, juveniles gradually reach the size of the adults and join the breeding group. After the breeding season, most adults die out and the cycle repeats. **b** The distribution of snout-vent length in each month demonstrates the annual cycle and the rapid growth from small-sized neonates and juveniles to large-sized adults

**Fig. 4:**
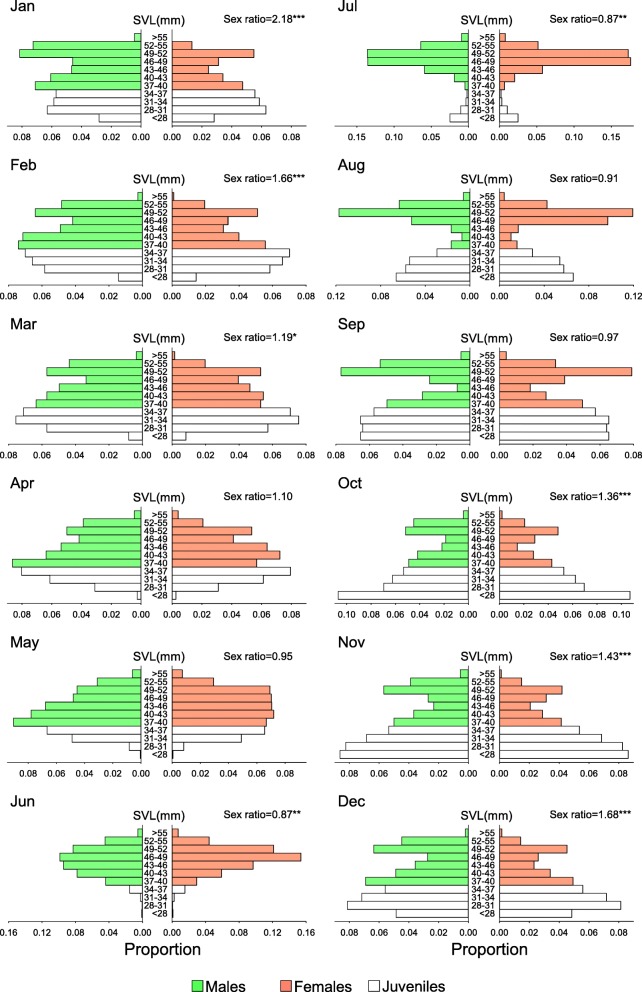
Monthly population pyramid and sex ratio in the *Takydromus viridipunctatus* population, taking from the mean of the eight-year capture data during 2006–2013. The majority of neonates begin to hatch in August, and reach the highest proportion of the population from September to December. In the meanwhile, some juveniles gradually reach the size of the adults since September, leading to the coexistence of two mature cohorts and forms a bimodal distribution of adult sizes from September to March. After June and July, the adults gradually die out, and the annual cycle repeats. At the beginning of breeding season in May, the population size of females outnumbered males, leading to the sex ratio (male/females) smaller than 1.0. However, the female-biased mortality during the breeding season leads to the decrease of females and increase of sex ratio. From October through April, the population becomes male-biased. However, penetration from juveniles to adults adjusts the sex ratio, and the annual cycle repeats. Asterisks showed the significance of sex ratio by using Chi-square test (*: *p* < 0.05, **:*p* < 0.01, ***:*p* < 0.0001)

Similar to the annual breeding tempo, the emergence of the young shows a seasonal peak. The number of juveniles is extremely low in June and July (Figs. 3a and 4), gradually increases from August on and peaks in October and November. Newly hatched neonates (the smallest-sized individuals) could make up the largest portion of the population among all size categories in these two months (Fig. 4), and even exceed 400 individuals being captured in a single night in November 2008.

**Fig. 5:**
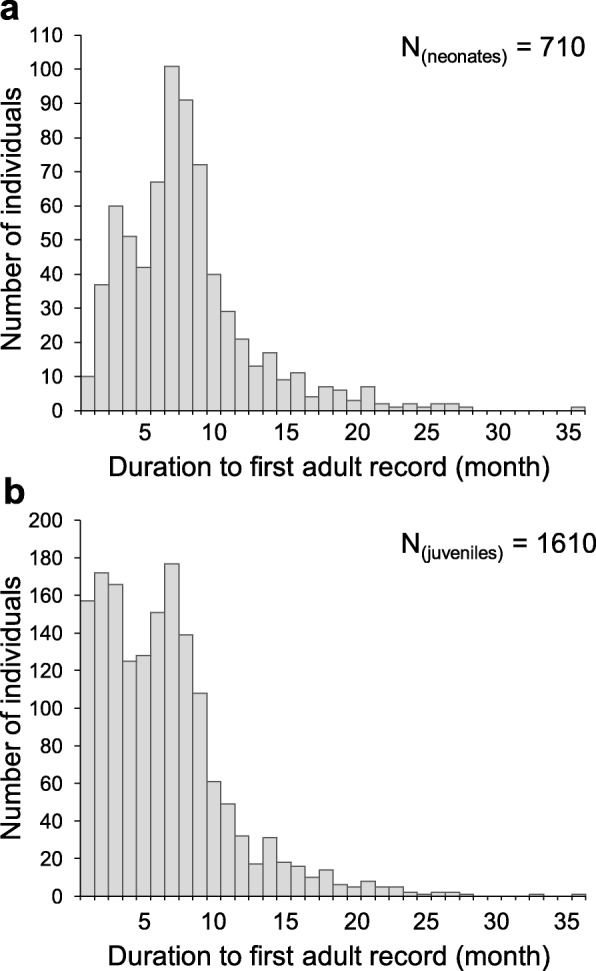
Fast growth and maturity of *Takydromus viridipunctatus* demonstrated by recapture records. **a** Among the 1610 individuals which were first captured as juveniles, over 91% of them (1465) reached sex maturity within one year. **b** Among a total of 710 newly hatched neonates (SVL smaller than 32.97 mm) which survived to adults, over 87% of them reached maturity within one year. Since the duration of two (or more) capture events relies on the premise that an individual should be successfully recaptured, the maturity speed could be under-estimated (faster, but not slower) in this figure

The neonates and juveniles remained the major component of the population from autumn, until the juveniles gradually reached sexual maturity in next spring (Figs. 3b and 4). From December to March, there existed a bimodal SVL size distribution among the adults (Fig. 4), indicating an older and a younger cohort coexisting in the population. From April on and through the breeding season, the young cohort caught up with the size of the older one, and joined the breeding group. Meanwhile, lizards from the older cohort gradually died out, returning SVL size distribution back to a unimodal distribution in summer, until neonates from the next generation emerged in autumn. These findings supported the implications induced from the fast and annual changes of the size distributions. Among a total of 1610 individuals which were first captured as juveniles and recaptured as adults, over 91% of them (1465) reached sex maturity within one year. More specifically, among 710 newly hatched neonates (SVL smaller than 32.97 mm) that survived to adulthood, > 87% of them reached maturity within one year (Fig. 5).

### Survival cost of reproduction

In survival analysis, the top three models, *φ*_sex*season_, *φ*_season_, and *φ*_sex + season_, were far better supported than the rest of the models according to the AICc and model weight (Table 1). The influence of season on survival was supported in all three top models and its significance was suggested when being compared to the null model of constant survival (LRT *φ*_season_ vs. *φ*_._: χ^2^ = 7.83, df = 1, *P* = 0.0051). In contrast, sex along was not a significant predictor of survival (LRT *φ*_sex_ vs *φ*.: χ^2^ = 1.74, df = 1, *P* = 0.1878), and it did not improve model fit when season was already incorporated (LRT *φ*_sex + season_ vs. *φ*_season_: χ^2^ = 1.84, df = 1, *P* = 0.1746). However, it is worth noting that the model with the interaction between sex and season (*φ*_sex*season_) was the best model. The interaction term marginally improved the model performance (LRT *φ*_sex*season_ vs. *φ*_season_: χ^2^ = 5.21, df = 2, *p* = 0.0740; *φ*_sex*season_ vs. *φ*_sex + season_: χ^2^ = 3.36, df = 1, *P* = 0.0667). Due to the similar fit of the best three, we estimated survival by averaging the three models.

**Fig. 6:**
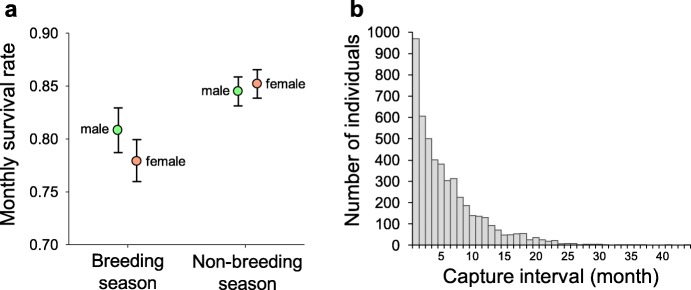
**a** Differences of monthly survival rates of males (green) and females (red) in the breeding and non-breeding seasons. Both sexes tend to have lower survival rate in the breeding season (*φ*_non-breeding female_: 0.8526, 0.8236–0.8776, *φ*_breeding female_: 0.7801, 0.7381–0.817; *φ*_non-breeding male_: 0.8454, 0.8170–0.8701, *φ*_breeding male_: 0.8091, 0.7646–0.8468). The increased mortality in the breeding season leads to a low survival rate during this period, and the significant decrease in females leads to a male-biased population structure in the non-breeding season. **b** Statistics of individuals with longest capture intervals. The high mortality rate leads to the short life span of the lizards: among the 4844 lizards ever being recaptured, only 11.44% (*N* = 554) survived for more than 12 months, and only 0.91% (*N* = 44) survived for more than 24 months. Short life span of Takydromus viridipunctatus demonstrated by recapture records

Given the marginal significance of the sex*season interaction, we separately estimated monthly survival for the males and females in the non-breeding season (*φ*_non-breeding male_: 0.8454, 0.8170–0.8701; *φ*_non-breeding female_: 0.8526, 0.8236–0.8776), as well as in the breeding season (*φ*_breeding male_: 0.8091, 0.7646–0.8468; *φ*_breeding female_: 0.7801, 0.7381–0.8170; Fig. 6a). Both sexes showed lower monthly survival in the breeding season than in the non-breeding season. The decline of the survival from the non-breeding season to the breeding season was more profound for females (females: 8.5% monthly decline; males: 3.63%). The survival estimates suggested that both sexes suffer a survival cost of reproduction, which was more severe for the females.

With such low monthly survival rate, our data of recaptured intervals showed that most of them experience a short life span as well. Among the 4844 individuals with at least one recapture record, only 554 (11.44%) were recaptured after 12 months; only 44 (0.91%) were recaptured after 24 months (Fig. 6b). Thus, a majority of the population had a short life span of no more than one year.

## Discussion

### A novel case of ICS

Our long-term data set confirmed the small and invariant clutch size (ICS) for this lizard population. Although ICS has been reported from more than 20 taxa in Squamata [12, 15], most cases occur in *Anolis* and gekkonid lizards [11]. This study presents one of the first confirmed cases in the Lacertidae family, which was deduced to have a VCS origin [12]. Furthermore, VCS was believed to be favored in a seasonal climate, whereas ICS usually occurs in tropical regions without obvious climate variation [12]. The case of ICS in *T. viridipunctatus*, from a subtropical monsoon region with a distinct seasonal climate, presents an opportunity to explore the interplay between seasonal environment and clutch size strategy.

Among all the nonexclusive hypotheses that have been proposed to explain the evolution of ICS, the “arboreal hypothesis” [15] best fits the case of *Takydromus* lizards. The specialized behavior to perch on the surface of grasses involves the extremely high necessity to keep their body mass not to exceed the supporting ability of these soft leaves (Fig. 1). On the other hand, the “predation hypothesis” [9, 15] may also partially explain the strategy in this lizard. A previous study [30] reported a high association between abundance of egrets and mortality of lizards, as well as that between abundance of shrike and kestrel and lizard tail loss and mortality. In the present study, the monthly survival rate yielded to an annual survival rate less than 0.1 (over 90% individuals died within one year), suggesting the low survival rate of this lizard compared to most other Squamata [21]. The high predation risk on adult lizards has been regarded as a selection for early reproduction [37, 38], which further leads to small maturity size, small clutch size because of body constraint, and multiple clutch per season [9, 15].

The third hypothesis predicts that ICS may have evolved to increase the size of the eggs, resulting in high-quality offspring. In some tropical regions, lizards tend to lay fewer eggs to produce larger hatchlings [9, 39, 40]. Population sizes in these regions are usually approaching the carrying capacity of the environment, yielding to the selection toward large and competitive hatchlings [9]. Obviously, this hypothesis does not fit the environment of the grass lizard, which utilizes open grasslands in early succession stage. Furthermore, we did not observe the obviously enlarged neonate size of *T. viridipunctatus* (SVL: 23.34 mm,) compared to other *Takydromus* species that lay large clutches (*T. amurensis*: 25.5 mm [41]; *T. hsuehshanensis*: 25 mm [42]; *T. septentrionalis*: around 25 mm [43]; *T. tachydromoides*: 24.9 mm [44]; *T. wolteri*: 21.8 mm [42]). Therefore, ICS as the consequence of K-selected strategy was not likely in this species. To compare the difference of clutch sizes among *Takydromus* species which inhabit different latitude/altitude or occupy different microhabitat niches is our great interest in the near future.

### High fecundity and low survival rate in high disturbance habitat

Our results showed multiple life history traits, such as seasonal breeding cycle, fast maturation, large reproductive potential, mass emergence of the young, and low monthly survival rate of *T. viridipunctatus*. All these traits represent the adaptation to the grassland of early succession stage under high and frequent disturbance.

Despite of the small and limited clutch size, the huge number of neonates hatched in late summer and fall (Figs. 3 and 4) indicated multiple clutches for a single female within a single breeding season. From the field data, we recorded over 200 recaptured females with more than two clutches within the same breeding season; the highest clutch number for a single female was five. These recapture records clearly demonstrate multiple clutches in this species. In laboratory, we also found that captive females are able to lay 2–4 clutches within two months from June to July (personal observations). Repeated producing clutches within a short time span has also been reported in several other *Takydromus* species [45–49], especially well noted in a captive case of *T. stejnegeri* which laid four clutches within 40 days [50]. Previous studies of lizard life history have suggested the classic continuum of reproductive pattern: small clutch size with multiple clutches within an extended breeding season, versus large clutch size only once in a short breeding season [9, 39, 51, 52]. Our results clearly indicate that this species is at the former end of this continuum.

*Takydromus viridipunctatus* is also characterized for its small body size and fast maturity schedule. After hatched in late summer, the young lizards represent a fast growth pattern in fall (Figs. 3 and 4). Since September, the snout-vent lengths of some juveniles begin to exceed the threshold of maturity size (36.19 mm for males and 37.89 mm for females). After the three-month winter with a low growing speed, most individuals of the young generation gradually catch up the last-year cohort, and join the breeding group in spring. Comparing to the data of other lizards from literature review (812 populations from 664 species) [9, 39, 52, 53], this is among the smallest species of which well-documented measurements have been conducted for large sample sizes, and is also the smallest compared to the 16 other well-studied species in the lacertid lizard family [51].

The final characteristics of life history traits in this lizard are the high mortality and short lifespan after maturity. As shown in the results, the annual survival rate was around 0.1 (0.8454^7^ × 0.8091^5^ = 0.1070 for males; 0.8526^7^ × 0.7801^5^ = 0.0946 for females) (Fig. 6a), and less than 1% individuals survived over two years (Fig. 6b). Such low survival rate is analogous to some small and highly productive *Anolis* lizards such as the well-studied *A. sagrei* [54]. Since annual species have been rarely reported in tetrapods [21], this species become one of the most short-lived species with solid capture-mark-recapture evidences.

### Unequal survival cost of reproduction between sexes

Our results showed a clear decline of survival rate during breeding season in both sexes. Moreover, the decline in females was significantly higher than that in males (Fig. 6a). The monthly survival rate between the sexes was similar in the non-breeding season from October to April (0.8454 in males, 0.8526 in female); however, these values distinctly decreased in the breeding season from May to September (0.8091 in males; 0.7801 in females). After the 5-month breeding period, the accumulative survival of males is 0.3467, while that of the females is 0.2889. Comparing to the 5-month survival rates in the non-breeding season (0.8454^5^ = 0.4318 in males, 0.8526^5^ = 0.4505 in females), the “survival cost of reproduction” is clearly revealed from both sexes, with especially high female-biased mortality during the breeding season.

Male-biased mortality has been commonly reported in a broad range of taxa [55–59], particularly in species with sexual dimorphism [60]. However, our results showed a female-biased mortality in this sexual dichromatic species. Several reasons might be responsible for this pattern. First, although the clutch size is small, the energetic and physical burden of pregnancy may be still severe for this species. As an ecological analog, *Anolis sagrei* also shows serious cost in physiology, locomotor performance, and survival in pregnant female despite the single-egg clutch [54, 61]. Secondly, pronounced male-male competition is less common in *Takydromus* lizards [30, 49, 62] that may result to the relatively weak cost for males. Thirdly, mating harassment from males to females may contribute to female-biased mortality in this dense population. Aggressive intersexual behavior reducing female survival has been suggested in many vertebrates [63–65], and also in lacertid lizards [65, 66]. During the breeding season, males bite females in the courtship behavior, forming more than 80% females with bite scars on their abdomen (Fig. 2b). Sexual conflict may partially explain this female-biased mortality in the breeding season.

## Conclusion

By using long-term monitoring with large sample size, we demonstrated the seasonal pattern and life history characteristics of a lacertid lizard, *Takydromus viridipunctatus*. Capture-mark-recapture program provided estimates on their life history traits including early maturity, high mortality, and short life span, and further represented a novel case of invariant clutch size (ICS) in a subtropical region. Occurrence of ICS in this lizard may be associated with the specialized habitat utility of grasslands, which limits their body mass; this trait is also well-adapted to the high predation risks in this environment. The elevated and female-biased mortality in the breeding season indicated the “cost of reproduction” of the lizard especially in females. Concluding these results, we demonstrated that the low clutch size could occur even in an *r*-selected environment of early succession stage.

## Supplementary information

**Additional file 1: Figure S1.** Monthly capture record of (a) adults; (b) juveniles; (c) recapture rates of all individuals; and (d) male/female composition (green/red) from May 2006 to August 2013. **Figure S2.** Sexual size dimorphism of *Takydromus viridipunctatus*.

## Data Availability

The datasets generated and/or analysed during the current study are available on Dryad.
