# Supplementary material for: Long-term monitoring reveals invariant clutch size and unequal reproductive costs between sexes in a subtropical lacertid lizard
Source: Zoological Lett. 2020 Jan 6;6:1. doi: 10.1186/s40851-019-0152-0 (PMC6945589; doi:10.1186/s40851-019-0152-0)
Supplement: Supplementary file 1 — Additional file 1: Figure S1. Monthly capture record of (a) adults; (b) juveniles; (c) recapture rates of all individuals; and (d) male/female composition (green/red) from May 2006 to August 2013. Figure S2. Sexual size dimorphism of Takydromus viridipunctatus. [file 40851_2019_152_MOESM1_ESM.docx]

Additional file 1

**Figure S1.** Monthly capture record of (a) adults; (b) juveniles; (c) recapture rates of all individuals; and (d) male/female composition (green/red) from May 2006 to August 2013.

**Figure S2.** Sexual size dimorphism of *Takydromus viridipunctatus*. (a) There was no difference in SVL between males (N=2375) and females (N=2129) (male_medium_=46.58 mm, female_medium_=47.38 mm; Wilcoxon rank-sum test: χ^2^=2.71, df=1, p=0.0996). (b) Body weight in males was significantly higher than that in females (b: male_median_=1.92 g, female_median_=1.83 g; Wilcoxon rank-sum test: χ^2^=61.49, p<0.0001). (c) The increase of mass difference between sexes following the increasing SVL was significant (multiple regression analysis with log-transformation in both weight and SVL: *R*^2^=0.7969, F_3,4500_=5886.72, p<0.0001; sex: *F*_1,4500_=6.56, p=0.0105; SVL: *F*_1,4500_=6538.44, p<0.0001; sex ×6SVL: *F*_1,4500_=9.69, p=0.0019).
